# Baltimore College of Dental Surgery

**Published:** 1858-10

**Authors:** 


*Baltimore College of Dental Surgery.*—We have recently received through Dr. Blandy, who is now in London, some very valuable contributions from Drs. Levison and Ballard, for the Museum of the above named institution, consisting of some hundreds of specimens of teeth, showing a great variety of abnormal developments and every description of pathological condition to which these organs are liable. There are also in the collection two sets of artificial teeth—one composed partly of ivory with human teeth mounted along the anterior part of the arches—the other, which is only designed for the upper jaw, is composed of ivory and English porcelain teeth. Both sets are clumsy and awkwardly constructed, showing but little mechanical skill and but a very imperfect knowledge of the art as practiced by dentists of the present day. They are tolerably fair specimens of mechanical dentistry as practiced forty or fifty years ago.

These contributions are very valuable and will be thankfully received by the Faculty of the College.

